# Key topographic parameters driving surface adhesion of *Porphyromonas gingivalis*

**DOI:** 10.1038/s41598-023-42387-5

**Published:** 2023-09-23

**Authors:** Steve Papa, Mathieu Maalouf, Pierre Claudel, Xxx Sedao, Yoan Di Maio, Hind Hamzeh-Cognasse, Mireille Thomas, Alain Guignandon, Virginie Dumas

**Affiliations:** 1 INSERM, SAINBIOSE U1059, Mines Saint-Etienne, Université Jean Monnet Saint-Étienne, 42023 Saint-Étienne, France; 2 GIE Manutech-USD, 20 Rue Benoît Lauras, 42000 Saint-Étienne, France; 3 Laboratory Hubert Curien, UMR 5516 CNRS, Jean Monnet University, University of Lyon, 42000 Saint-Étienne, France; 4 Ecole Centrale de Lyon, CNRS, ENTPE, LTDS, UMR5513, ENISE, Univ Lyon, 42023 Saint-Étienne, France

**Keywords:** Biomedical engineering, Microbiology, Nanoscale materials, Ultrafast lasers, Implants, Bioinspired materials, Mechanical properties, Metals and alloys

## Abstract

Dental implant failure is primarily due to peri-implantitis, a consequence of bacterial biofilm formation. Bacterial adhesion is strongly linked to micro-/nano-topographies of a surface; thus an assessment of surface texture parameters is essential to understand bacterial adhesion. In this study, mirror polished titanium samples (Ti6Al4V) were irradiated with a femtosecond laser (fs-L) at a wavelength of 1030 nm (infrared) with variable laser parameters (laser beam polarization, number, spacing and organization of the impacts). Images of 3-D topographies were obtained by focal variation microscopy and analyzed with MountainsMap software to measure surface parameters. From bacteria associated with peri-implantitis, we selected *Porphyromonas gingivalis* to evaluate its adhesion on Ti6Al4V surfaces in an in vitro study. Correlations between various surface parameters and *P.* *gingivalis* adhesion were investigated. We discovered that Sa value, a common measure of surface roughness, was not sufficient in describing the complexity of these fs-L treated surfaces and their bacterial interaction. We found that Sku, density and mean depths of the furrows, were the most accurate parameters for this purpose. These results provide important information that could help anticipate the bacterial adhesive properties of a surface based on its topographic parameters, thus the development of promising laser designed biofunctional implants.

## Introduction

Titanium-6aluminum-4vanadium (Ti6Al4V) is mainly used as a biomaterial for dental implant devices due to its excellent physicochemical properties and high biocompatibility with host tissues^1,2^. Osseointegrated dental implants are safe with high survival rates at about 90% and minimal marginal bone resorption in the 10 years following implantation^3^. However, once exposed to the oral environment, implants are subject to mucositis, which is a reversible inflammatory condition that affects the soft tissues surrounding a dental implant. It typically occurs within a few weeks to months after the implant placement. During the initial phase, the body recognizes the dental implant as a foreign object, leading to a natural inflammatory response. If proper oral hygiene is not maintained, plaque and bacteria can accumulate around the implant, causing inflammation of the mucosal tissue surrounding it. At this stage, the condition is still reversible, and with prompt treatment and improved oral hygiene, the inflammation can be controlled and resolved. If left untreated, mucositis can progress to peri-implantitis, one of the most important unsolved biological complications in recent implantology^4,5^. Peri-implantitis is a destructive inflammatory lesion that affects the surrounding soft and hard tissues with a loss of supporting bone^3^ and is responsible for up to 47% of failed oral implants^6^. Implants that can reduce bacterial adhesion would help to improve the outcomes of dental surgeries, especially considering an ever increasing risk of infection as bacteria gain resistance to antibiotics^7^. Currently, there is no strong evidence to suggest an effective implant surface treatment to develop efficient and reliable antibiofilm solutions and avoid infections such as peri-implantitis^8^.

Surface topographic characteristics of implants can significantly impact the attachment of oral bacteria, implying that surface nano- and microroughness are strongly and positively correlated with bacterial adhesion^9–11^. In this regard, many innovative surfaces have been developed with the idea of reducing adhesion strength between bacterial strains and a substrate by tailoring surface nanostructures^12–14^. It is crucial for future studies to follow a standardized approach when evaluating the topography-based antibacterial activity of different surfaces. This will allow for direct comparison of results between different research groups. To achieve this, the methods used for surface physicochemical characterization and antibacterial assay should be consistent and well defined to ensure accurate and quantitative comparisons^11^. Since bacterial cell walls typically have similar mechanical properties, understanding the underlying antibacterial mechanism based on surface topography can lead to the development of a universal measure of antibacterial efficacy based on surface topography. This measure could be applied to surfaces of various shapes and dimensions, from rigid to flexible structures. Developing a systematic theory that considers all influencing factors of the mechanism will enable the classification of surfaces according to their antibacterial efficiency. Therefore, the aim of the present study was to identify topographic parameters directly linked to bacterial adhesion and evaluate their degree of relevance for industrial texturing of biomaterials such as dental implants.

Among several surface treatments, ultrafast laser texturing has emerged as a powerful and versatile surface engineering process for dental implant applications, presenting higher biomineralization activity and stimulated osseointegration^15–17^, while also inhibiting bacterial adhesion on implant surfaces^18,19^. Surface features may significantly modify the properties of surfaces that either allow or limit bacterial adhesion^20–22^. Femtosecond laser (fs-L) enables the development of different types of laser-induced nanostructures, such as laser-induced periodic surface structures (LIPSS) with linear, radial, or azimuthal organization^23,24^. The fs-L is a very valuable innovative approach due to its simplicity, the lack of clean room facility requirements, and the ability to perform processing in airborne environments. Therefore, the fs-L texturing process is a good candidate for implementation in an industrial setting for dental implant treatment.

To assess surface topography, many studies use only the roughness parameter (Ra or Sa) to provide a surface description^10,25–27^. However, the real surface geometry is more complicated and many other parameters could be used to provide a more accurate description^28^. This paper investigates three groups of surface roughness parameters defined as amplitude, hybrid, and spacing parameters.

The oral microbiome is particularly complex and relies on polymicrobial synergies of multiple spatially constrained microbial communities that interact by providing attachment sites, crossfeeding and maintaining synergistic/antagonistic growth relationships^29^. An alteration of this balance, related either to host or bacterial community factors, results in dysbiosis. The latter favors the emergence of periodontopathogens whith virulence factors that affect other species and initiate inflammation. The resulting inflammation reinforces dysbiosis by exerting selective pressure on microbial communities and providing inflammophilic periodontopathogens with nutrients derived from altered tissue. In turn, dysbiosis fuels inflammation, creating a vicious cycle that promotes periodontal disease. In the context of peri-implantitis, the biofilm formation process plays a crucial role in the development of this inflammatory condition around dental implants. The beginning of biofilm formation is characterized by the adsorption of proteins and molecules from the oral environment onto the implant surface, forming an initial conditioning film, and providing a favorable environment for bacterial attachment. Subsequently, early colonizing bacteria, known as pioneer species, take advantage of this conditioning film to adhere and establish themselves on the implant surface. These pioneer bacteria play a significant role in shaping the biofilm community. Their presence and interactions with the implant surface create a foundation for further bacterial colonization and biofilm growth. The most effective periodontal pathogens, members of the red complex (*Porphyromonas gingivalis*, *Treponema denticola*, *Tannerella forsythia*), are widely responsible for implant failure^30,31^. For its clinical interest within pathologies such as rheumatoid arthritis, we focused our study on *P. gingivalis*. When we consider the upper part of a dental implant, we can assume that perfectly flat medical titanium is offering a fair defense against infection. Surface engineering based approaches are considered as a highly competitive alternative to obtain comparable performance for delivering repellent factors more safely and efficiently. Nevertheless, surface characterization should be more precise since bacterial adhesion relies not only on Sa, which is mostly described in the literature as a determining parameter. Therefore, we have correlated *P.* *gingivalis* adhesion with amplitude (areal arithmetic mean height (Sa), maximal height of the pits (Sp), maximal height of the valleys (Sv), skewness values (Ssk), kurtosis value (Sku)), hybrid (root mean square of slopes (Sdq), developed interfacial area ratio (Sdr)) and spacing parameters (density of furrows, mean furrows depth) in an in vitro study.

## Material and methods

Mirror polished titanium alloy samples of Ti6Al4V were purchased from Goodfellow (Huntingdon, UK). These Ti6Al4V samples were dimensioned in squares with a surface area measuring 1 cm^2^, and a thickness of 1 mm by the supplier prior to shipment.

### fs-L irradiation

Titanium alloy samples were textured at the GIE Manutech-USD platform (France). Two workstations composed of a 1030 nm fs-L from Amplitude Systems (France) coupled with a galvo scanner from Scanlab (Germany) and 3D-XYZ translation stages from Aerotech (Germany) were employed to produce linear LIPSS, azimuthal LIPSS, and radial LIPSS.

Linear LIPSS were generated on titanium surfaces using a Tangor HP fs-L linearly polarized with a pulse duration of 500 fs. The repetition rate was defined at 100 kHz and focused thanks to a 100-mm f-theta lens. A line with an interspot distance of 5 µm in X direction was obtained with a constant translation speed of 0.5 m/s, repeated in Y direction with the same distance of 5 µm resulting in a recovering rate of around 84% in both directions and an estimated local accumulation of 32 pulses.

The azimuthal and radial LIPSS were produced using a Satsuma HP fs-L linearly polarized with a pulse duration of 300 fs (pulse duration slightly shorter but not sufficiently relevant to disturb the LIPSS formation compared to the previous setup). The repetition rate was defined at 500 kHz and focused with a f-theta lens of 50 mm. Respectively, radial and azimuthal polarizations were used for azimuthal and radial LIPSS using a dedicated s-wave plate from Workshop of Photonics (Lithuania). For these patterns, a pixel-by-pixel texturing strategy was chosen, where pixels were arranged in X and Y so as to generate different lattices of different sizes following two different patterns with square and hexagonal shapes. The detailed laser process parameters are summarized in Table 1.

**Table 1:** Laser parameters used to create linear, azimuthal and radial LIPSS.

LIPSS	Pulse energy (µJ)	Beam polarization	Impacts		Distance between pulses		F-theta (mm)
			Number per cavity	Alignment	Δx (µm)	Δy (µm)	
L	1.07	Linear	32	Straight	5	5	100
A1	0.78	Radial	25	Square	14	14	50
R1	0.78	Azimuthal	25	Square	14	14	50
A2	0.78	Radial	25	Square	13	13	50
R2	0.78	Azimuthal	25	Square	13	13	50
A3	0.78	Radial	25	Hexagonal	6	10	50
R3	0.78	Azimuthal	25	Hexagonal	6	10	50
A4	0.78	Radial	25	Hexagonal	8	13	50
R4	0.78	Azimuthal	25	Hexagonal	8	13	50
A5	0.78	Radial	5	Hexagonal	6	11	50
R5	0.78	Azimuthal	5	Hexagonal	6	11	50

For bacterial adhesion tests, laser textures were distributed in 4000 × 100 µm bands alternating with 100 µm of untextured titanium for a local control.

### Surface morphology

Scanning electron microscopy (SEM) was used to visualize and characterize the different laser induced patterns. A Tescan VEGA3 SB (Brno Czech Republic) electron microscope was used, operating at 15 kV and using the secondary electron detector.

### Surface topography

Surface parameters were measured on images taken with an optical 3-D microcoordinate system by InfiniteFocusIF G4, ALICONA (Graz, Austria), which uses the focus variation microscopy technique. The measurements were made with a 1000× magnification, 400-nm lateral resolution and 10-nm vertical resolution. Data topographies were analyzed with Mountains Map^®^ 8.2 software to measure surface roughness parameters focusing on the following: Sa, Sp, Sv, Ssk, Sku, Sdq, Sdr, density of furrows, and mean furrows depth. Definitions of surface parameters are stated by ISO 25178 standards.

### Bacterial strains and culture conditions

*Porphyromonas gingivalis* ATCC 33277 strain was isolated on Brucella Blood Agar under anaerobic conditions using the Genbox system (Biomerieux); then cultured 4 d in Schaedler broth with mineral oil (Sigma M5904) at a ratio of 1:20 (mineral oil:medium) at 37 °C. 1 mL of broth was incubated over titanium samples for 48 h at 37 °C under anaerobic conditions.

### Fluorescent bacteria labeling

After 48 h of incubation, medium was removed; bacteria were stained with calcein AM (Invitrogen; C3099) at 10 µg mL^−1^ for 20 min at 37 °C; then washed with PBS and fixed with 10% formalin (Sigma HT501128) for 40 min at room temperature; washed with PBS; and kept in PBS at 4 °C until observation.

### Image acquisition and analysis

Fluorescent microscopy (Zeiss LSM 800 Airyscan, Oberkochen, Germany) was used to image stained bacterial samples. Image analysis was performed with ImageJ by measuring mean fluorescence in 30 areas (7.53 mm^2^) per condition and making a percent ratio with local polished control. Fixed bacteria were dehydrated in graded ethanol solutions (70, 80, 90 and 100%), air dried at room temperature and analyzed with SEM at 5 kV with a secondary electron detector.

### Statistical analysis

All statistical analyses were performed using GraphPad Prism 8 software. Bacterial data are presented as mean ± SEM. Statistical analyses were performed using *t*-test. Graphical representation of the different correlations shows Spearman's rank-order correlation and associated corrected *p*-value; correlation is considered as significant if corrected *p*-value < 0.05.

## Results

**Figure 1:**
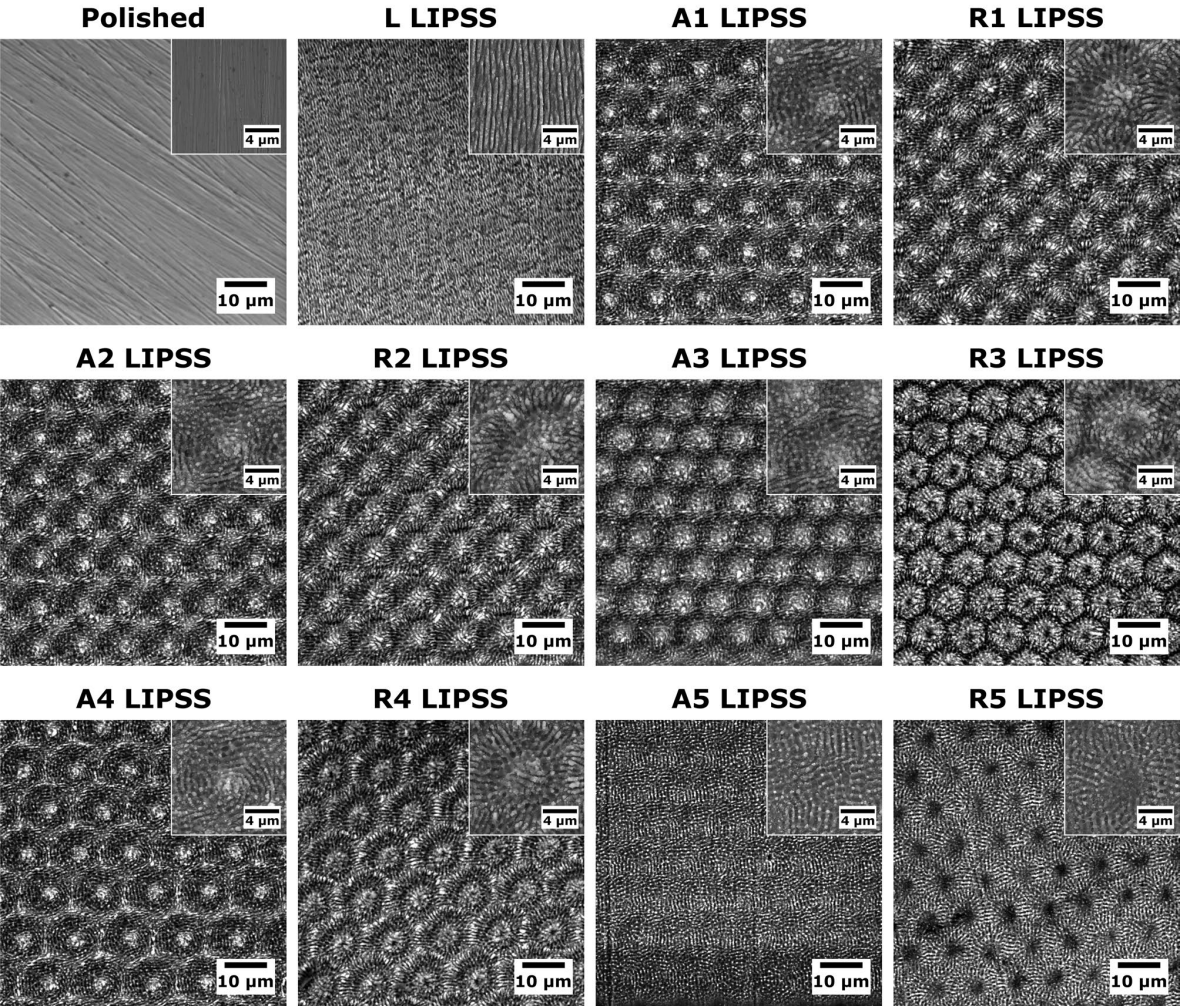
Surfaces visualization and associated laser parameters. SEM images of polished and LIPSS-textured Ti6Al4V surfaces.

IR fs-L irradiation of Ti6Al4V creates highly ordered/periodic texturing called IR LIPSS, as presented on SEM images (Fig. 1). With LIPSS direction being perpendicular to the laser beam polarization, linear LIPSS (L LIPSS) were obtained with linear polarization, azimuthal LIPSS were obtained with radial polarization (A1 to A5 LIPSS), and radial LIPSS with azimuthal polarization (R1 to R5 LIPSS), as observed in SEM images.

**Figure 2:**
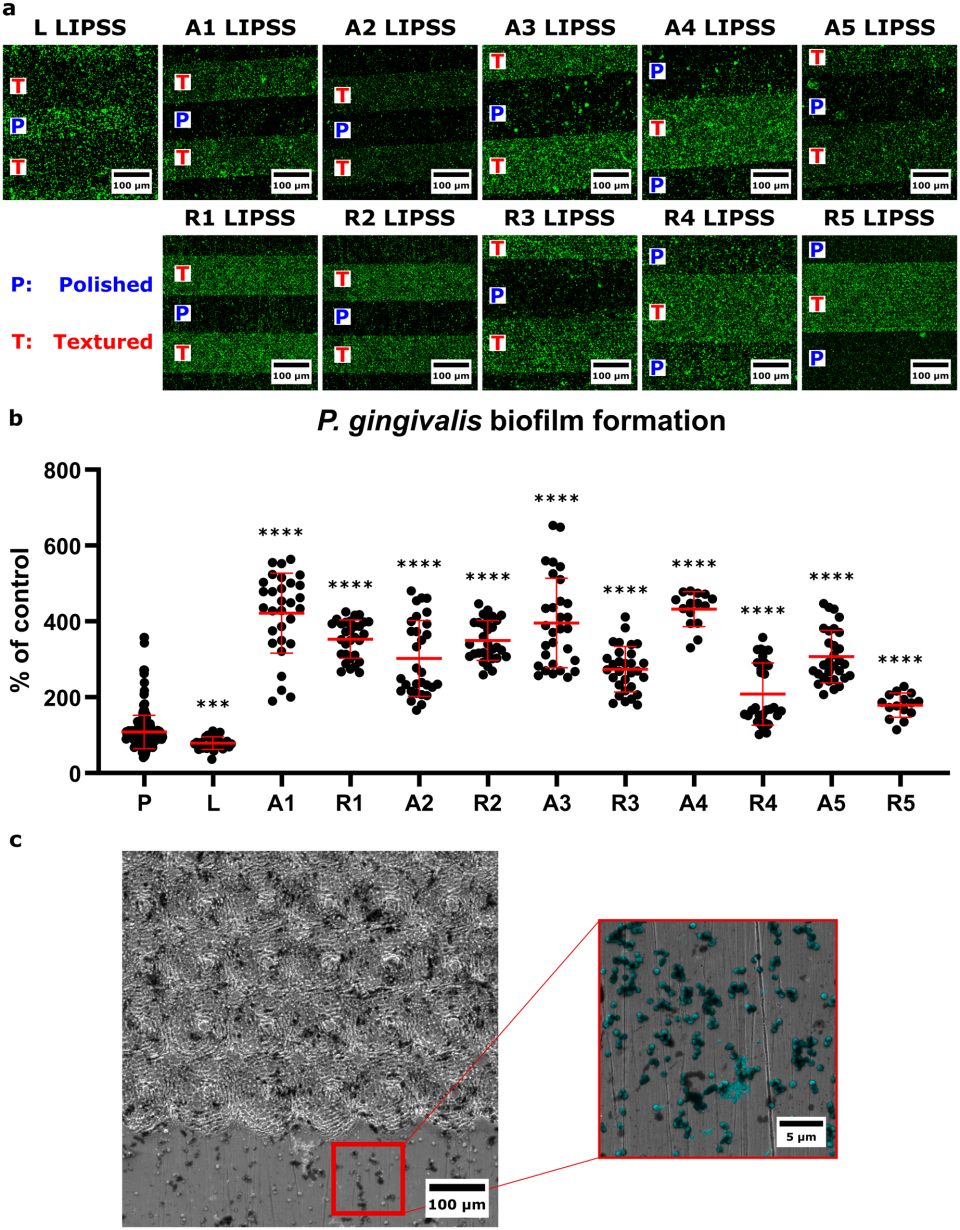
Evaluation of *P. gingivalis* adhesion on the different surfaces. (**a**) Representative fluorescence images of calcein stained *P. gingivalis* 48 h postseeding on partially textured samples. (**b**) Graph presenting the fluorescence quantification as mean ± SEM. n = 30 area/group; *t*-test compared to polished control; ***p = 0.0003; ****p < 0.0001. (**c**) SEM images of *P. gingivalis* on partially textured surfaces.

Imaging of stained *P. gingivalis* after 48 h of culture on partially textured samples (Fig. 2a) allowed the quantification of bacterial adhesion over LIPSS with a ratio compared to the adjacent control (internal reference) on bordering polished surfaces (Fig. 2b). Results show a decrease in *P.* *gingivalis* adhesion by 30% on linear LIPSS compared to the polished control; whereas all fluorescence quantifications on azimuthal and radial LIPSS demonstrated an increase in *P.* *gingivalis* adhesion from 70% up to 324%. Adherent *P.* *gingivalis* are presented over textured/polished surfaces in SEM images (Fig. 2c).

**Figure 3:**
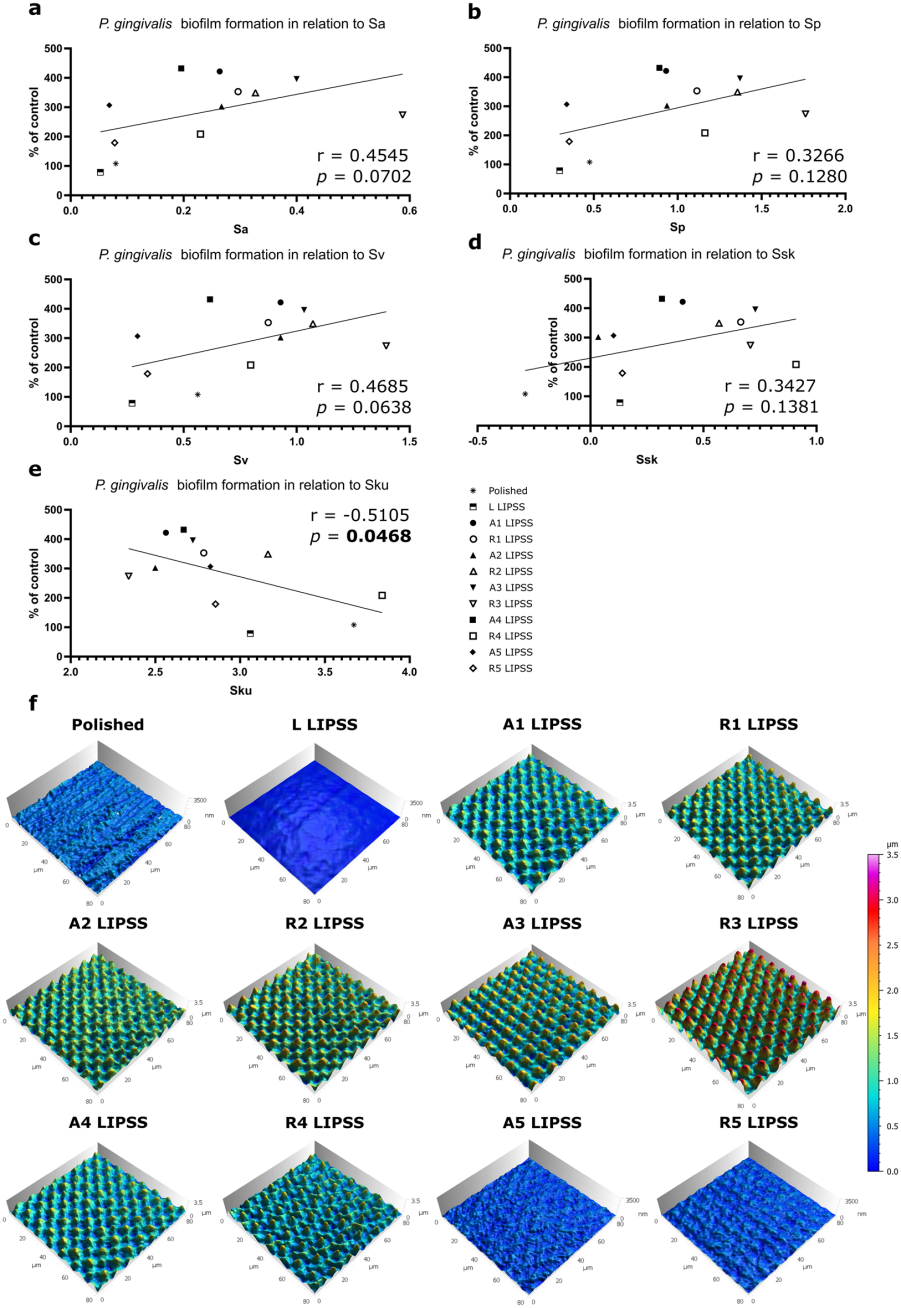
Surface topographies and associated parameters. Graphical representation of the correlation between *P. gingivalis* biofilm formation and Sa (**a**), Sp (**b**), Sv (**c**), Ssk (**d**) or Sku (**e**) with the Spearman's rank-order correlation and associated p-value. (**f**) 3-D representation of surface topographies.

Correlations between surface parameters and bacterial adhesion, presented in Fig. 3, revealed the importance of some amplitude parameters such as Sa (Fig. 3a), Sp (Fig. 3b), Sv (Fig. 3c), Ssk (Fig. 3d) and Sku (Fig. 3e) in the bacterial adhesion of *P.* *gingivalis*. Representations of the textured surfaces presented in Fig. 3f allow the visualization of 3-D surface topographies. Bacterial adhesion of *P.* *gingivalis* was significantly correlated only with Sku (p = 0.0468); whereas the correlation with Sa, the roughness parameter commonly selected to evaluate bacterial adhesion, was only a tendency (p = 0.0702).

**Figure 4:**
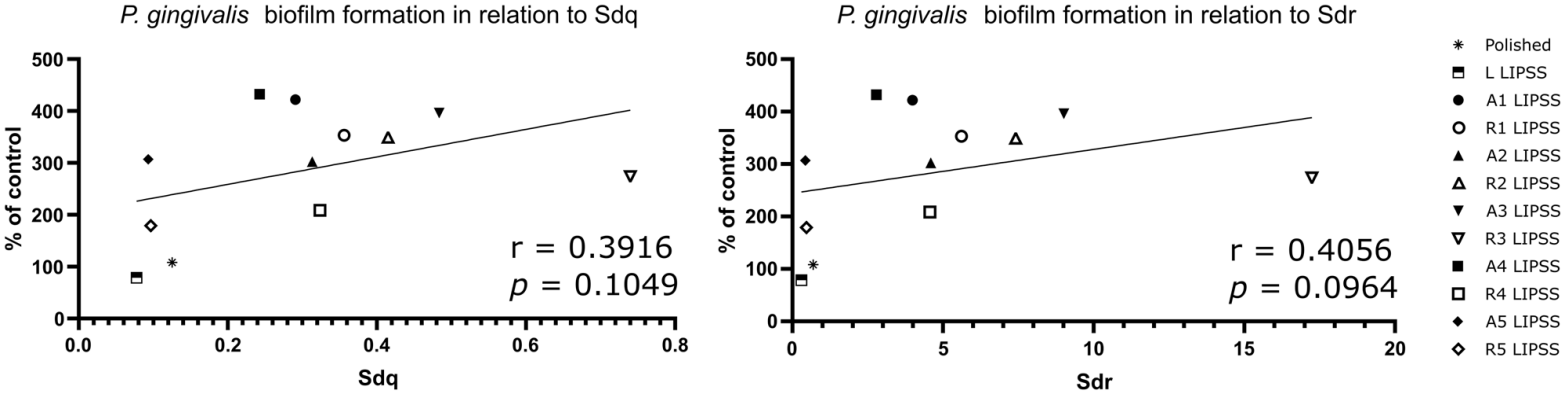
Hybrid parameters. Graphical representation of the correlation between *P. gingivalis* adhesion and Sdq or Sdr with the Spearman's rank-order correlation and associated *p*-value.

The following analysis is focused on hybrid parameters, presented in correlation with *P.* *gingivalis* adhesion in Fig. 4. Surface level evolution was evaluated with Sdq and developed surface was measured with Sdr. The polished controls as well as textured surfaces with the lowest laser irradiation (linear; S8; S17) showed a Sdq close to 0.1 (± 0.025) and a Sdr close to 0.5 (± 0.19) due to the low complexity of these surfaces compared to the other textured surfaces, which showed 2—6 times higher Sdq and 4–25 times higher Sdr than the polished controls. Nevertheless, *P.* *gingivalis* adhesion was not significantly related to any of these hybrid parameters.

**Figure 5:**
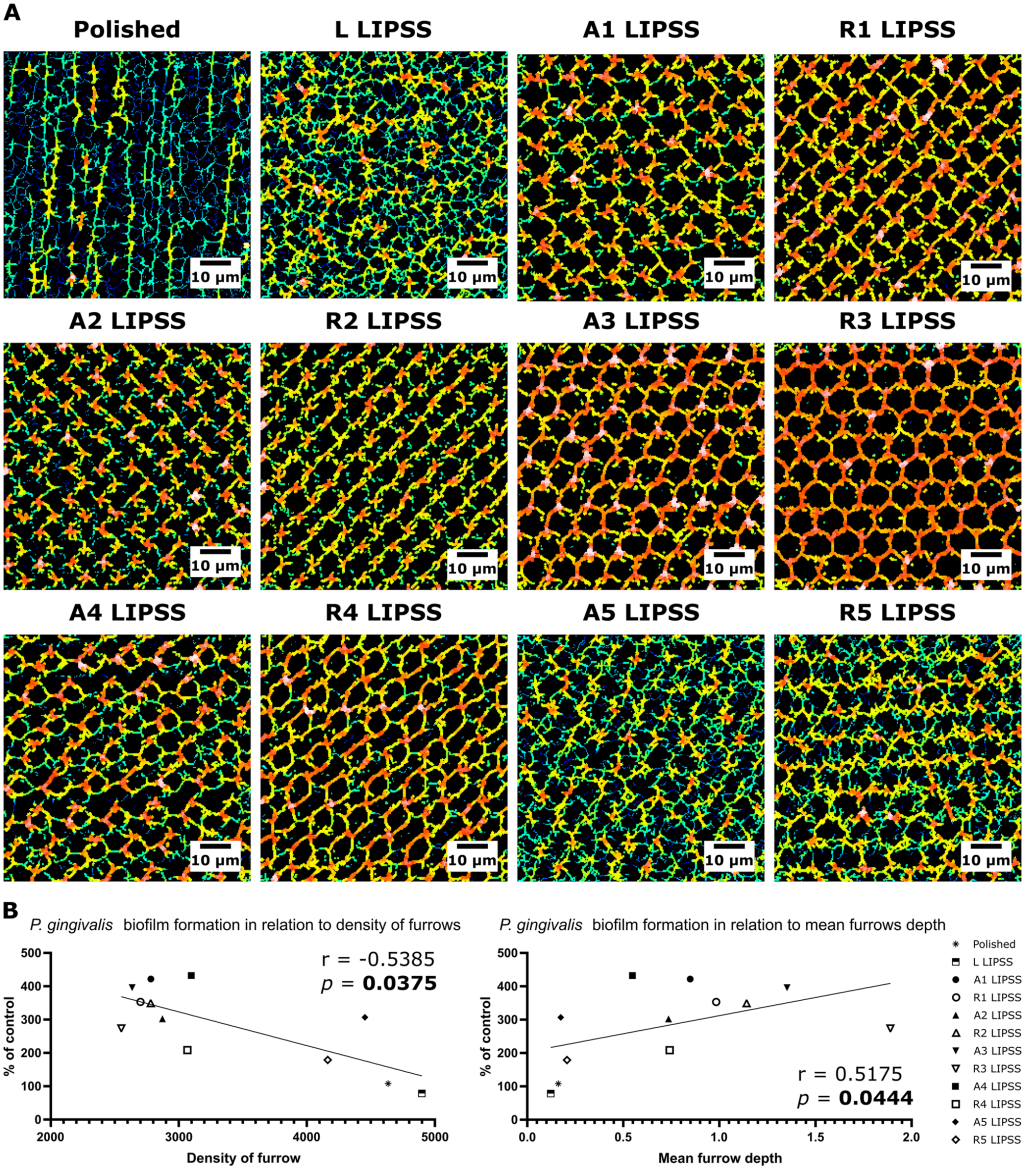
Furrows characterization. (**A**) Qualitative rendering of the furrows. (**B**) Graphical representation of the correlation between *P. gingivalis* adhesion and density of furrows or mean furrows depth with the Spearman's rank-order correlation and associated *p*-value.

MountainsMap software provided qualitative renderings that were also obtained from Fourier transforms to the profile height function (Fig. 5A). These renderings simulated furrows and arranged contour lines along the surface that represent qualitative aspects of the surface texture. Graphical representations of *P.* *gingivalis* adhesion (Fig. 5B) show a negative correlation with the density of furrows but a positive correlation with the mean furrows depth.

## Discussion

In terms of tissue engineering, dental implants require different properties from top to bottom; as the upper part needs to be textured to increase gingival tissue adhesion^32^, while the largest part being implanted in the jaw must present pro-osteogenic textures^22^. As a versatile tool, fs-L is perfectly suited for this purpose by offering an alternative of multiple texturing along the entire implant surface. However, special attention must be paid to the surface texture parameters to avoid increased bacterial adhesion.

Both 2-D and 3-D forms can be used to calculate surface texture parameters. Not long ago, 2-D profile analysis was prevalent; but in recent decades, 3-D surface analysis is more relevant for science and engineering applications^28^. 3-D roughness parameters are more suitable for surface analysis since they are evaluated for a complete area instead of a single profile.

Regarding bacterial adhesion evaluation, we can confirm the antiadhesive properties of IR LIPSS towards *P.* *gingivalis*, as demonstrated in a previous study^32^. Concerning every azimuthal and radial LIPSS evaluated, we can only assume that these texturings increased *P.* *gingivalis* surface adhesion; nevertheless, it is not possible to determine the precise impact of each texture. However, the increase in bacterial adhesion is not as important on all those textured surfaces. The characterization of fs-L textured surfaces is more complicated than just LIPSS orientation; therefore, correlations between bacterial adhesion and individual roughness parameters are of special interest.

Concerning amplitude parameters, roughness (Ra or Sa) is the commonly studied surface characteristic^8,33^. Here, Sa is correlated with *P.* *gingivalis* adhesion in a nonsignificant manner. This trend (*p* = 0.0702) is in agreement with previous studies concerning this correlation^25,34^. Moreover, the Sv value, which represents the maximal height of the valleys, also showed a tendency (*p* = 0.0638) in correlation with bacterial adhesion. SEM analysis of bacteria adherent to the texturing revealed that deep valleys that are larger than a single bacterium diameter may retain bacteria, resulting in increased bacterial adhesion (data not shown). The absence of correlation with Sp is coherent as the height of pits is not a determinant factor for the amount of bacteria retained by the texture.

The Ssk values represent the degree of bias of the roughness shape, meaning the height distribution (peaks and pits) around the mean plane. This does not rely on the maximal level of peaks, pits, or the spatial distribution of LIPSS; therefore, the absence of correlation with bacterial adhesion seems coherent.

Sku is a way to describe the global relief of a surface; more precisely, it symbolizes the shape of the distribution of profile points^28^. A value of Sku < 3 characterizes a surface presenting relatively few high peaks and low valleys; whereas a value of Sku > 3 illustrates a topography with many high peaks and low valleys. Herein, Sku is significantly correlated with *P.* *gingivalis* adhesion; meaning that an increased number of deep furrows leads to enhanced bacterial adhesion to the surface.

Concerning hybrid parameters, indicators of the slopes of the surface for both Sdq and Sdr show that no determinant correlation has been identified with bacterial adhesion.

There is a strong correlation between spatial topography parameters and bacterial adhesion. The critical factor is the surface of contact between a single bacterium and the substrate^8,11^. A rise in mean furrows depth as well as a decrease in the mean density of furrows result in an increase in the contact surface, which explains a higher bacterial adhesion as the bacteria will fit easier in the furrows. A special focus must be done on both reducing the depth and increasing the amount of furrows on fs-L textured surfaces to reduce contact surface and thus bacterial adhesion, as suggested in the literature^11,35,36^.

Even if different works evaluate the correlation between bacterial adhesion and roughness parameters, it is quite hard to make a comparison of different studies, given that topographic characterization differs depending on the resolution of the measuring device used. Atomic force microscopy describes nanoscaled topographies; here with multifocus microscopy, we describe it on a microscale. In terms of bacterial adhesion, nanorugosity will directly impact single bacterium comportment in a positive or negative way; however, a microroughness will increase mature biofilm formation^35^. Therefore, there is a real interest in developing multiscale measuring tools^37^.

We could not rule out the possibility that modifying surface chemistry might impact bacterial adhesion to surfaces with different topographies. According to Florian et al., the polished sample surface is expected to have a naturally occurring oxidation layer of about 10 nm, while the linear LIPSS covered surface would have an oxidized layer of about 50 nm^38^. Concerning radial and azimuthal LIPSS, the oxidation layer thicknesses are assumed to be slightly above or below 50 nm, depending on the number of laser pulses per cavity. Since our study is centered on low spatial frequency LIPSS, it is reasonable to assume that the different textures in our study have oxide layers of similar thickness^38^. The variation in thickness should be minimal, and thus its role in bacterial adhesion is deemed to be insignificant.

Results showed correlations between *P.* *gingivalis* adhesion and some surface parameters, which is of major interest for dental implants. Otherwise, many bacterial strains are involved in peri-implantitis; it would be really interesting to confirm these studies with other bacteria of the red complex or primary colonizers such as *Streptococcus mutans*^6,39,40^. An application to other biomaterials could be envisaged and would necessitate evaluation of the adhesion of nosocomial bacterial strains such as *Staphylococcus aureus* or *Escherichia coli*. Nevertheless, according to certain literature^11,32^ and this present study, there is a crucial importance of generating a texturing of a smaller size than a single bacterium; meaning that results with *P.* *gingivalis*, which is approximately 600 nm in diameter, could be valid for every bacterium of a larger size. Also, as suggested by Linklater et al., the mechanical properties of bacterial cell walls are similar between strains; therefore, an understanding of all influencing factors of the topography based bactericidal mechanism could lead to a precise description of mechanobactericidal surface patterns^11^. A recent study has shown that differences in surface topography not only impact bacterial adhesion, but also modify decontamination with powders and ultrasounds, as well as subsequent fibroblast growth^41^. A similar study could be carried out on LIPSS from the present study. Previous study from our team showed that radial LIPSS have an interest regarding the osteogenesis surrounding dental implants^22^; nevertheless, regarding bacterial adhesion, the linearity of the LIPSS should be prioritized. As shown in a previous study, it is more important to focus on reducing the spatial period of the LIPSS in order to reduce bacterial adhesion^32^. With the aim of reducing bacterial adhesion to dental implants surfaces, particular interest should be given to the minimization of spatial spacing in the axis perpendicular to the linear orientation of the texturing and which should be prioritized. To go further in the evaluation of the impact of such parameters in bacterial adhesion, a study focused on surfaces presenting similar Sa with various spacing parameters should be led. However, it is obvious that Sa parameter evaluation is definitely not sufficient for bacterial attachment assessment and probably also cell tissue adhesion. A more global and more precise description of surface topographies would be promising for the design of future biomaterials such as dental implants.

## Conclusions

Laser processing enables the creation of reproducible micro- and nanotextures (LIPSS) on titanium-based surfaces in a one-step process. By fine tuning the fs-L parameters, precise control of both micro- and nanotopographies can be achieved. This study shows that for dental implant applications, it is recommended to focus on linear LIPSS as the sole antiadhesive texturing approach. In assessing bacterial adhesion, relying solely on Sa (a common parameter used to anticipate adhesion) may not be sufficient. Additional attention should be given to spatial parameters, such as the density and mean depth of furrows, as well as the Sku of the surface, as they are strongly associated with *P. gingivalis* adhesion. While this study demonstrates promising results, further research is needed to investigate the effect of different bacteria on ultrafast laser surface texturing and its potential implications for bacterial adhesion anticipation.

### Fi-index tool

This manuscript has been checked with the Fi-index tool and obtained a score of 1.5 for the first author and respectively 0.30 and 0.65 for the last authors (value on the date 21/08/2023 according to Web of Science^®^)^42,43^. The Fi-index tool aims to ensure the quality of the reference list and limit any autocitations.

## Data Availability

Raw datasets may be available from the corresponding author on reasonable request**.**
